# Ginsenoside Rb3 alleviates smoke‐induced lung injury via the H19/miR‐29b‐3p/HGMB1/TLR4 signalling pathway

**DOI:** 10.1111/jcmm.15844

**Published:** 2021-02-01

**Authors:** Yan Tan, Danxiong Sun, Juan Chen, Rufang Li, Shenglan Wang

**Affiliations:** ^1^ Department of Pulmonary and Critical Care Medicine The First People’s Hospital of Yunnan Province Kunming China

**Keywords:** CSE, ginsenoside, HGMB1, inflammation, lncRNA‐H19, miR‐29b‐3p

## Abstract

The over‐activation of inflammation is involved in the pathogenesis of smoke‐induced lung injury (SILI), while Rb3 treatment may alleviate smoke‐induced lung injury by down‐regulating the expression of H19, a regulator of miR‐29b expression. Moreover, HMGB1 is an important mediator of inflammation. Therefore, in this study, we set up an animal model of SILI and treated it with Rb3 to study the effect of Rb3 on the treatment of SILI and the involvement of H19/miR‐29b/HMGB1/TLR4 signalling. SILI mice treated with Rb3 before H&E staining and TUNEL assay were conducted to observe the pathological damages and status of apoptosis in each group. Real‐time PCR, Western blot, computational analysis and luciferase assays were utilized to establish the signalling pathway involved in the pathogenesis of SILI and the action of Rb3 treatment. Rb3 treatment alleviated pathological changes in the lungs while decreasing the levels of W/D ratio and cell apoptotic index. H19 was validated to sponge miR‐29b‐3p, while HMGB1 mRNA was validated to be a target gene of miR‐29b‐3. As a result, a signalling pathway of H19/miR‐29b‐3p/HMGB1 was established. Cell viability was evidently reduced after 72 hours of treatment with CSE, but the treatment of Rb3 elevated the expression of H19 and HMBG1 in the presence of CSE. Also, CSE‐induced inhibition of miR‐29b‐3p expression was restored by Rb3. The findings of this study collectively demonstrated that Rb3 exhibited its therapeutic effect during the treatment of SILI via modulating the H19/miR‐29b‐3p/HMBG1 signalling pathway.

## 1 INTRODUCTION

As a leading contributor of global mortality, cigarette smoke (CS) can cause pulmonary disorders such as lung inflammation, which is featured by the elevated expression of inflammatory cytokines such as IL‐1β and TNF‐α, which in turn result in neutrophil activation.^1^, ^2^, ^3^ And in a recent study, Rb3 was reported to exert protective properties against smoke‐induced lung injury (SILI) by increasing cell survival while reducing oxidative stress and inflammatory reactions.^4^ LncRNAs can exert its inhibitory effect upon miRNA by acting as a miRNA sponge. For example, the level of serum miR‐29b‐3p was reduced by up‐regulated H19 and the down‐regulation of H19 inhibited cell growth, migration while promoting along with cell apoptosis.^5^ MiR‐29b‐3p, on the other hand, can bind to the 3’ UTR of high‐mobility group box 1 (HMGB1) mRNA and reduce HMGB1 protein expression, and HMGB1 is an important mediator of inflammation.^6^ Therefore, the above data collectively suggested that miR‐29b‐3p may exert a critical effect on HMGB1 expression. In addition, the secretion of HMGB1 from liver cells substantially decreased the severity of inflammation in hepatocytes.^7^ Furthermore, Rb3 treatment may down‐regulate the expression of H19, a regulator of miR‐29b expression.^5^, ^8^

## 2 MATERIAL AND METHOD

Mice groups were established as follows: a Control group (N = 15), a CS group (N = 15), an Rb3 group (N = 15) and a CS + Rb3 group (N = 15). The dose of Rb3 treatment was 20 mg/kg via oral administration after being dissolved in 0.5% carboxymethyl cellulose (CMC). Lung tissue samples and alveolar lavage fluid (ALF) were collected from the mice in different groups for subsequent analyses. The SILIC mice models were established by putting the mice into a Perspex chamber with a size of 30 L and exposing the mice to a 2 hours cigarette smoke twice a day for 4 consecutive weeks. The right lung of each mouse was collected and processed into a 4 μm thick section following a routine H&E staining, and the samples were observed underneath an IX‐70 inverted light microscope. Moreover, the semi‐quantitative lung injury score of lung tissues was calculated.

A549 and H460 cells were cultured in DMEM and then transfected with H19 siRNA, miR‐29b‐3p mimics or HMGB1 siRNA using Lipofectamine 2000. Meanwhile, the cells were also established as an untreated group, a CSE group, an Rb3 group (with 10 μmol/L Rb3 added to culture) and a CSE + Rb3 group. The expression of according genes in the above‐described cell groups was subsequently measured by real‐time PCR and Western blot.

An online miRNA targeting tool was used to identify HMGB1 and miR‐29b‐3p as potential target genes of miR‐29b‐3p and H19. A549 and H460 cells were co‐transfected with wild‐type or mutant H19/HMGB1 in conjunction with miR‐29b‐3p mimic/negative controls, and the luciferase activity was evaluated at 48h after the transfection.

The relative expression of H19, miR‐29b‐3p mRNA and HMGB1 mRNA was measured following routine real‐time PCR procedure with the cycle threshold approach, and the expression of HMGB1 and TLR4 proteins in cell and tissue samples was measured using a routine Western blot assay. The cell apoptosis profiles were measured using a TUNEL assay kit following kit directions and the cell viability was measured using MTT assay kits following kit instructions.

For statistical analysis, SPSS 23.0 software was utilized to carry out all statistical analyses and the significance level was set to 0.05.

## 3 RESULTS

**Figure 1:**
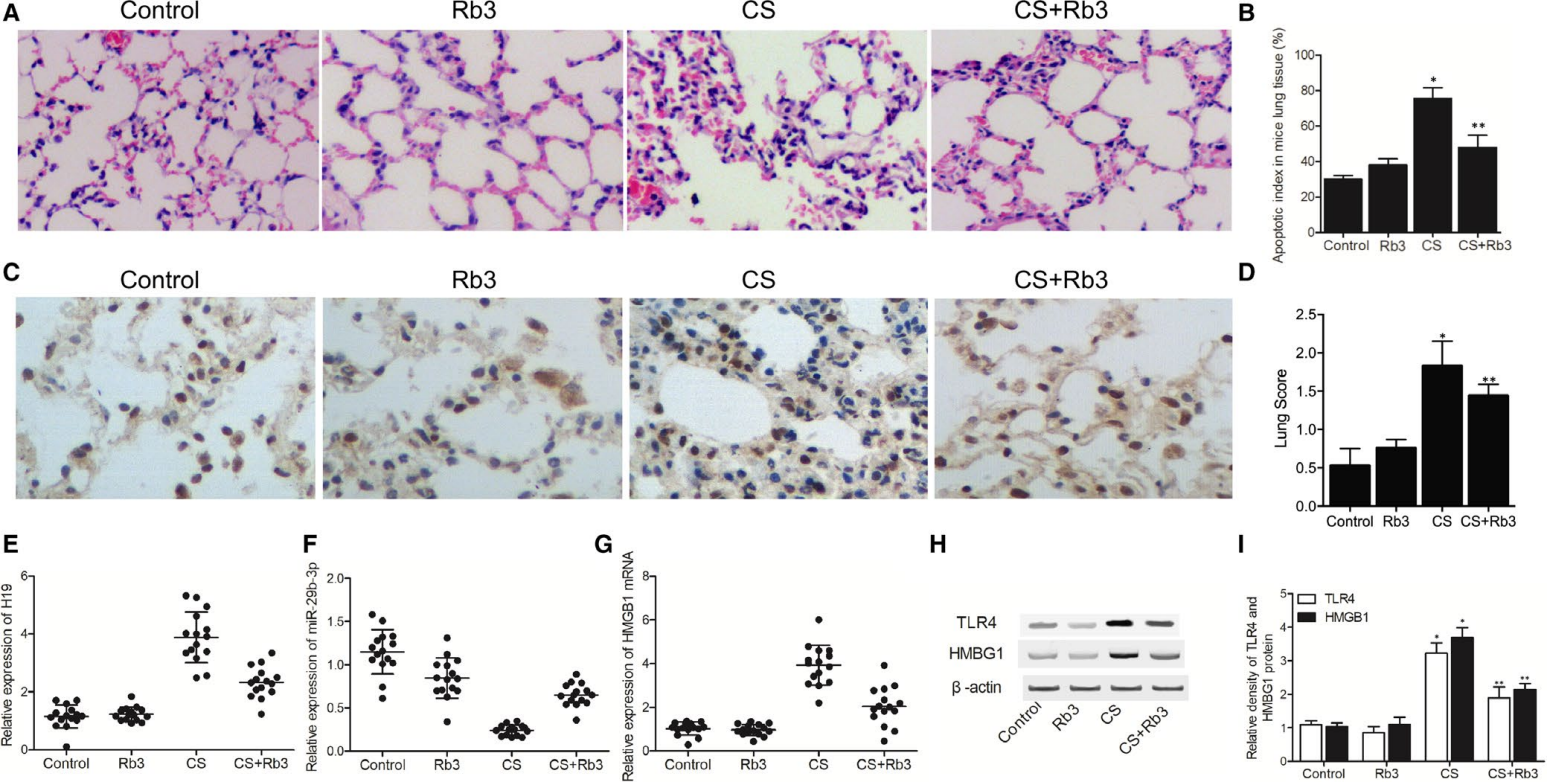
Rb3 reduced lung inflammation and apoptosis, and regulated the expression of SILI‐related genes in mice models. A, H&E staining of tissue samples collected from the Control group, Rb3 group, CS group and CS + Rb3 group; B, TUNEL assay of tissue samples collected from the Control group, Rb3 group, CS group and CS + Rb3 group; C, Cell apoptotic rate in the Control group, Rb3 group, CS group and CS + Rb3 group (*P value < 0.05, vs control group; **P value < 0.05, vs CS group); D, Semi‐quantitative lung injury score in the Control group, Rb3 group, CS group and CS + Rb3 group(*P value < 0.05, vs control group; **P value < 0.05, vs CS group). E, Relative expression of H19 in the Control group, Rb3 group, CS group and CS + Rb3 group; F, Relative expression of miR‐29b‐3p in the Control group, Rb3 group, CS group and CS + Rb3 group; G, Relative expression of HMGB1 mRNA in the Control group, Rb3 group, CS group and CS + Rb3 group; H, Western blotting showed the relative expression of HMGB1 and TLR4 proteins in the Control group, Rb3 group, CS group and CS + Rb3 group; I, Relative density of HMGB1 and TLR4 protein bands in the Control group, Rb3 group, CS group and CS + Rb3 group (*P value < 0.05, vs control group; **P value < 0.05, vs CS group)

Compared with the control group, lung tissues in the CS group presented evident alveolar wall thickening with severe cell inflammations, and Rb3 partly obstructed this change (Figure 1A). Also, the cell apoptotic rate was significantly aggravated in CS group, which was alleviated by Rb3 treatment (Figure 1B,C). Meanwhile, the semi‐quantitative lung injury score was markedly lowered by Rb3 treatment in CS mice (Figure 1D). Therefore, Rb3 could exhibit positive effects in the treatment of SILI.

**Figure 2:**
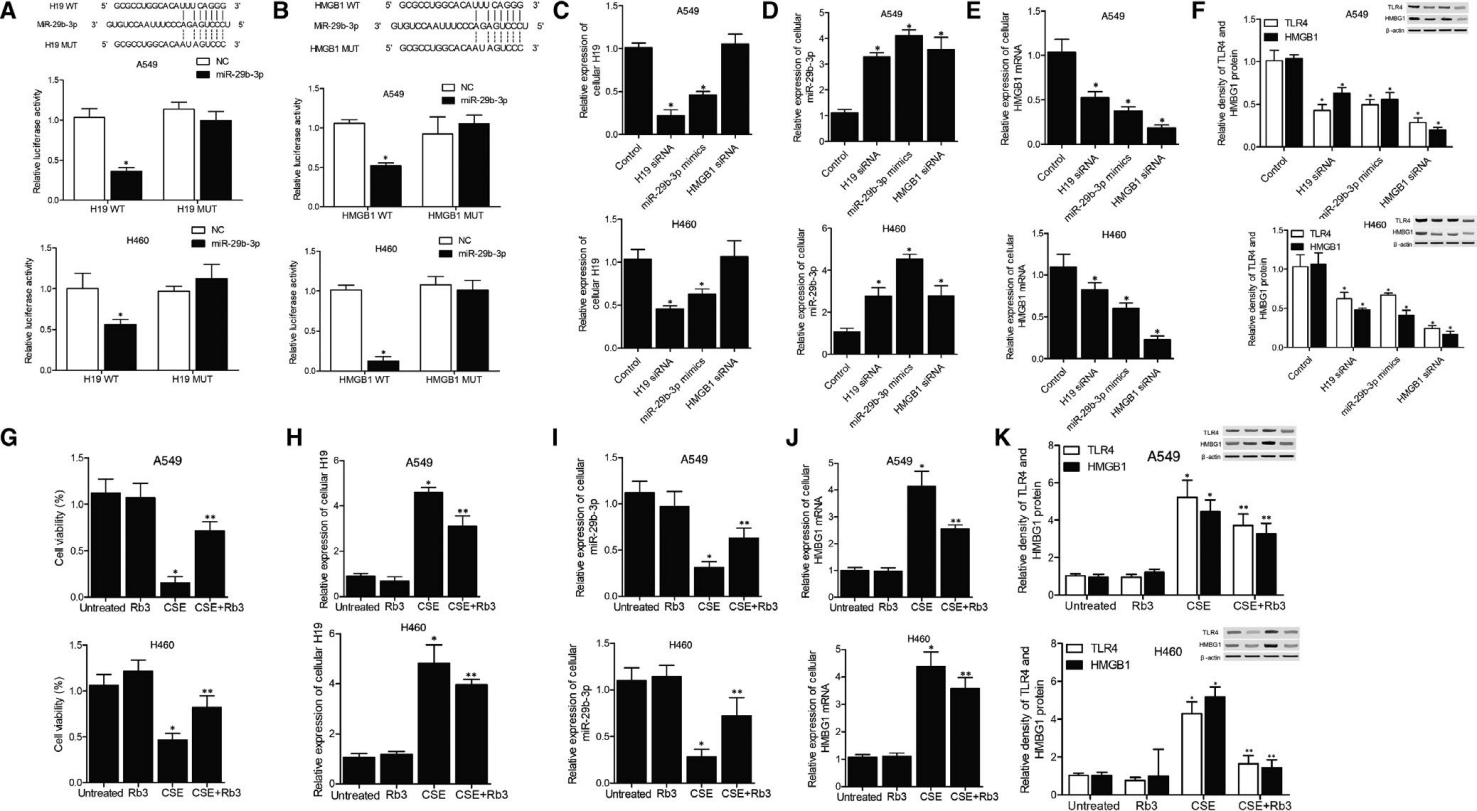
The establishment and validation of the H19/miR‐29b‐3p/HMGB1 signalling pathway in A549 and H460 cell models. A, Computational analysis between miR‐29b‐3p sequence and H19 sequence, and relative luciferase activity of wild‐type/mutated H19 in A549 and H460 cells transfected with miR‐29b‐3p or control miRNAs (*P value < 0.05, vs control miRNAs); B, Computational analysis between miR‐29b‐3p sequence and HMGB1 mRNA sequence, and relative luciferase activity of wild‐type/mutated HMGB1 mRNA in A549 and H460 cells transfected with miR‐29b‐3p or control miRNAs (*P value < 0.05, vs control miRNAs); C, Relative expression of H19 in A549 and H460 cells transfected with control miRNAs, H19 siRNA, miR‐29b‐3p mimics and HMGB1 siRNA, respectively (*P value < 0.05, vs control group); D, Relative expression of miR‐29b‐3p in A549 and H460 cells transfected with control miRNAs, H19 siRNA, miR‐29b‐3p mimics and HMGB1 siRNA, respectively (*P value < 0.05, vs control group); E, Relative expression of HMGB1 mRNA in A549 and H460 cells transfected with control miRNAs, H19 siRNA, miR‐29b‐3p mimics and HMGB1 siRNA, respectively (*P value < 0.05, vs control group); F, Western blotting and relative density of HMGB1 and TLR4 protein bands in A549 and H460 cells transfected with control miRNAs, H19 siRNA, miR‐29b‐3p mimics and HMGB1 siRNA, respectively (*P value < 0.05, vs control group). G, The viability of A549 and H460 cells divided into an untreated group, an Rb3 group, a CSE group and a CSE + Rb3 group (*P value < 0.05, vs untreated group; **P value < 0.05, vs CSE group); H, Relative expression of H19 in A549 and H460 cells divided into an untreated group, an Rb3 group, a CSE group and a CSE + Rb3 group (*P value < 0.05, vs untreated group; **P value < 0.05, vs CSE group); I, Relative expression of miR‐29b‐3p in A549 and H460 cells divided into an untreated group, an Rb3 group, a CSE group and a CSE + Rb3 group (*P value < 0.05, vs untreated group; **P value < 0.05, vs CSE group); J, Relative expression of HMBG1 mRNA in A549 and H460 cells divided into an untreated group, an Rb3 group, a CSE group and a CSE + Rb3 group (*P value < 0.05, vs untreated group; **P value < 0.05, vs CSE group); K, Western blotting and relative density of HMGB1 and TLR4 protein bands in A549 and H460 cells divided into an untreated group, an Rb3 group, a CSE group and a CSE + Rb3 group (*P value < 0.05, vs untreated group; **P value < 0.05, vs CSE group)

Compared with the control group, the expressions of H19 (Figure 2E), HMGB1 mRNA (Figure 1G), HMGB1 protein (Figure 1H,1I) and TLR4 protein (Figure 1H,I) were all up‐regulated while the expression of miR‐29b‐3p (Figure 1F) was inhibited in CS group. Meanwhile, Rb3 treatment evidently alleviated the dysregulation of these genes and proteins, suggesting the possible therapeutic role of Rb3 in the treatment of SILI.

By conducting a computational analysis, potential binding sites of miR‐29b‐3p were respectively located on H19 (Figure 2A) and HMGB1 mRNA (Figure 2B), and subsequent luciferase assays showed evidently reduced relative luciferase activity in A549 and H460 co‐transfected with mutant H19 and miR‐29b‐3p (Figure 2A) or mutant HMGB1 mRNA and miR‐29‐3p (Figure 2B), indicating a potential regulatory relationship between H19/HMGB1 and miR‐29b‐3p.

Moreover, in the subsequent validation experiments in A549 and h460 cells, the relative expression of H19 (Figure 2C) was suppressed while the relative expression of miR‐29b‐3p (Figure 2D) was increased in the presence of H19 siRNA or miR‐29b‐3p. And the transfection of H19 siRNA, miR‐29b‐3p mimics or HMGB1 siRNA all resulted in evident inhibition of HMGB1 mRNA (Figure 2E), HMGB1 protein (Figure 2F) and TLR4 protein (Figure 2F). Therefore, a signalling pathway of H19/miR‐29b‐3p/HMGB1 was established.

Cell models were established to validate the effect of Rb3 and the possible underlying mechanisms. Accordingly, the cell viability in the CSE group was evidently reduced but partially restored by Rb3 treatment (Figure 2G). And the expressions of H19 (Figure 2H), HMBG1 mRNA (Figure 2J), HMBG1 protein (Figure 2K) and TLR4 protein (Figure 2K) were all evidently elevated in A549 and H460 cells treated with CSE for 72h, while the expression of miR‐29b‐3p (Figure 2I) should the opposite trend as compared with H19. However, Rb3 partly obstructed the dysregulations expression of these SILI‐related genes and proteins. Therefore, it can be concluded that Rb3 exhibited its therapeutic effect during the treatment of SILI via modulating the H19/miR‐29b‐3p/HMBG1 signalling pathway.

## 4 DISCUSSION

Ginseng has been utilized as an important drug in traditional Chinese medicine for thousands of years and exerts significant effects in the treatment of respiratory diseases.^9^ As a key active ingredient of ginseng, Rb3 plays an anti‐inflammatory role to delay the injuries induced by cigarette smoke.^4^ In this study, Rb3 was observed to alleviate the pathological changes in SILI mice lungs by decreasing the semi‐quantitative lung injury score, and Rb3 decreased the up‐regulated expression of H19 and HMGB1 mRNA/protein while increasing the down‐regulated expression of miR‐29b‐3p in SILI mice. Therefore, Rb3 is possibly involved in the pathogenesis of SILI via modulating the expression of these SILI‐related genes.

MiR‐29b participates in the regulation of cell cycle progression, apoptosis as well as the metastasis of tumour cells,^10^, ^11^ and miR‐29b‐3p can reduce the growth and viability while promoting apoptosis of lung cancer cells.^12^ In this study, H19 was validated as a sponge of miR‐29b‐3p, while HMGB1 mRNA was validated as a target gene of miR‐29b‐3p, thus establishing a signalling pathway of H19/miR‐29b‐3p/HMGB1.

Upon release from cells, HMGB1 can activate the signalling pathways downstream of Toll‐like receptors, a superfamily of proteins with the ability to promote the synthesis of numerous inflammatory mediators as well as cytokines.^13^, ^14^, ^15^ In this study, cell viability was evidently reduced when treated with CSE for 72 hours. The elevated levels of H19 and HMBG1 mRNA/protein in CSE‐treated cells were reduced by the treatment of Rb3. Also, the inhibited expression of miR‐29b‐3p in CSE‐treated cells was increased by Rb3.

## 5 CONCLUSION

Taken together, our results suggested that Ginsenoside Rb3 can prevent SILI via H19/miR‐29b‐3p/HGMB1/TLR4 signalling pathway. Therefore, Ginsenoside treatment might be used as a new strategy for treating SILI.

## CONFLICT OF INTEREST

The authors confirm that there are no conflicts of interest.

## AUTHOR CONTRIBUTION

**Yan Tan:** Conceptualization (lead); Investigation (equal); Project administration (lead); Writing‐original draft (equal). **Danxiong Sun:** Formal analysis (equal); Investigation (equal); Software (equal). **Juan Chen:** Formal analysis (equal); Investigation (equal); Validation (equal). **Rufang Li:** Investigation (equal); Methodology (equal). **Shenglan Wang:** Conceptualization (supporting); Project administration (supporting); Writing‐original draft (equal); Writing‐review & editing (lead).

## Data Availability

The data that support the findings of this study are available from the corresponding author upon request.
